# Internalized Stigma among Patients with Mental Illness Attending Psychiatric Follow-Up at Dilla University Referral Hospital, Southern Ethiopia

**DOI:** 10.1155/2018/1987581

**Published:** 2018-01-23

**Authors:** Biksegn Asrat, Alem Eskeziya Ayenalem, Tenaw Yimer

**Affiliations:** ^1^Department of Psychiatry, School of Medicine, College of Medicine and Health Science, University of Gondar, Gondar, Ethiopia; ^2^Department of Psychiatry, School of Medicine, College of Health Science, Dilla University, Dilla, Ethiopia; ^3^Department of Public Health, College of Health Science, Debre Markos University, Debre Markos, Ethiopia

## Abstract

**Background:**

This study tried to reflect evidences regarding internalized stigma and assessed risk factors of internalized stigma.

**Objective:**

It aims to assess the magnitude, domains, and covariates of internalized stigma among patients with mental illness in southern Ethiopia.

**Methods:**

The study was carried out by using a cross-sectional study design at Dilla University Referral Hospital (DURH). A total of 317 patients with mental illness were involved. Internalized stigma was measured using Internalized Stigma of Mental Illness (ISMI) scale. Data analysis was done using SPSS version 20. Descriptive statistics and logistic regression were done.

**Result:**

The prevalence of internalized stigma was 32.1% among people living with mental illness in Dilla University Referral Hospital. Being female, medication nonadherence, and lack of family support were factors independently associated with internalized stigma.

**Conclusion:**

The results of this study found an intermediate prevalence of internalized stigma among people living with mental illness in Dilla University Referral Hospital (DURH). It revealed how much antistigma campaigns are so much important to tackle internalized stigma among people living with mental illness. Incorporating counseling and structured therapy played an important role in maximizing their quality of life.

## 1. Introduction

People living with mental illness (PLWMI) are facing double problems: their illness and the stigma. Stigma is perceived because most Ethiopian societies believed that mental illness is a punishment by God and linked with evil possession [1]. Stigma is unjustifiably held belief, feeling, and behavior towards people with mental illness (PWMI) [2, 3]. Stigma results from a process by which certain individuals (groups) unjustifiably are rendered shameful, excluded, and discriminated [3]. Stigma experienced by patients with mental illness (PWMI) is classified into three ways [2]: perceived stigma: what the individual thinks about societies' beliefs about the stigmatized group; experienced stigma: actual discrimination experienced by mentally ill individuals; self-stigma: a product of the internalization of public stigma. Internalized stigma is internalizing and accepting public stigma in adherence to the three concepts of stigma to sustain his/her life [2–4].

The prevalence of internalized stigma among psychiatric patients is high. Study findings from Iran revealed 40% prevalence of internalized stigma among people with mental illness [5]. A community-based study in California, USA, found 36% prevalence of internalized stigma among people living with severe mental illness [6]. Similarly, a study result from the University of Maryland, USA, reported a 35% prevalence of internalized stigma among this group of people [7]. Another institution-based study from Nigeria, Federal Neuro Psychiatric Hospital, revealed a 22.5% prevalence of internalized stigma [8]. A study in Addis Ababa, Ethiopia, found that 97.4% of people with mental illness experienced at least one internalized stigma item [9].

Internalized stigma affects the quality of life in several ways. It worsens mental illness and leads to social exclusion, inability to participate in important life activities, and poor tendency to seek treatment which at last hampers one major dimension of quality of life (QoL) [2, 5, 10, 11]. Strong internalized stigma is associated with lower level of self-esteem, self-efficacy, and poor recovery of people living with mental illness; finally, they become unemployed [7, 12, 13]. Stigma affects patients' help-seeking behavior, patients' adherence towards their medication, and the rehabilitation process negatively [14]. It affects the lives of people living with mental illness regardless of sociodemographic difference and it is negatively linked with treatment adherence, hope, self-esteem, and empowerment of these groups of people [15].

Type and severity of mental illness, education, the status of support, and religion are major influencing factors of internalized stigma. Due to internalized stigma held by people with mental illness (PWMI), there is a poor treatment behavior, higher prevalence of disability, and economic burden [11, 16].

In Ethiopia, mental illness is believed to be caused by evil spirit possession, evil attack, and punishment by God for one's wrongdoing [14, 17]. A recent study that was done in southwest Ethiopia reported 25.1% prevalence of internalized stigma among people with mental illness [18]. Further, this study found a lower risk of stigma among those with high income, educated, and active religious individuals.

Internalized stigma is one of the factors that increase mental illness-related morbidity and mortality. It affects help-seeking behavior, adherence to prescribed medications, and influence for substance use. Furthermore, it increases the intention to commit suicide [19].

The possible factors for the high risk of stigma are ignorance, perceived fear of injury, actual (perceived) absence of treatment, and cultural misconceptions (lack of information) about the nature of mental illness, associating the illness with the supernatural explanation [3, 14, 16, 18].

Although some effort tried to reduce stigma against PWMI, stigmatization, and discrimination towards PWMI, it is still persistent. A study in the capital city of Ethiopia on outpatient attendees with schizophrenia reported that almost all patients encountered internalized stigma [20]. In Ethiopia, especially in this study area, there is widespread mental illness associated with deep-rooted stigma towards people with mental illness (PWMI) [19, 21].

Very few scientific evidences are found in Ethiopia regarding internalized stigma against mentally ill individuals. It is still not an area of interest for researchers and this study aimed to generate a scientific evidence of how much the problem is deep-rooted. Understanding the magnitude and sources of a stigma that is related to mental illness is very crucial. The study has great relevance for stakeholders to tackle the problem because understanding the magnitude of the problem and modifiable risk factors is imperative. It will assist in designing prevention strategies and giving direction to minimize deleterious effect of internalized stigma on lives of people with mental illness (PWMI). It will also allow evaluating institution-based mental health care interventions in relation to antistigma activities in southern Ethiopia. The aim of this study was to determine the magnitude, domains, and covariates of internalized stigma among patients with mental illness in Dilla University Referral Hospital (DURH), South Ethiopia.

## 2. Methodology

### 2.1. Study Design and Setting

The study was an institution-based cross-sectional survey carried out at Dilla University Referral Hospital (DURH). Dilla University Referral Hospital (DURH) is the only teaching and referral hospital in Gedeo Zone. Patients from Gedeo Zone come to the hospital to get psychiatric care. The hospital provides its services for around 847.434 people in its catchment area. The service is provided by more than 12 mental health professional specialists (M.S. degree holders in mental health). The team manages a variety of patients with mental illness (PWMH) referred from health post, health centers, and district hospitals all over the region.

### 2.2. Participants

The sample size was determined using single population proportion formula. The parameters used to calculate the sample size included a proportion of 25% prevalence of internalized stigma, taken from similar study results in Jimma University Teaching Hospital, Ethiopia [18]. Furthermore, a 95% confidence (*Z* = 1.96) interval at 5% margin of error was taken. Accordingly, the minimum sample size was (*n*) = 288. The sample size was also adjusted to compensate for nonresponse rate of 10%. And the final sample size was 317.

(1) n=Z2p1−Pd2,n=1.962×0.251−0.250.052.

*n* = 288, when 10% nonresponse rate was assumed to be *n* = 317.

Respondents were screened to assess their eligibility for the study using Clinical Global Impression (CGI) scale. The scale assesses the severity of the patients' mental illness, remission of their illness, and efficacy index of therapeutic and drug side effects. New patients were screened only for the severity of their illness. Using this scale and their clinical experience, the psychiatric nurses identified the eligible respondents. Patients who were severely psychotic, incoherent, and too disorganized to engage in the interviews of the study were excluded. Therefore, patients included in the study were only those who were above 18 years old and rated with at least a less severe state of mental illness, on improvement and good efficacy index by the psychiatry nurses.

### 2.3. Measures

The Internalized Stigma of Mental Illness (ISMI-29) tool was utilized to measure internalized stigma (internal consistency reliability coefficient of alpha = 0.90) [22]. The tool was validated and has been used in several studies in Ethiopia [9, 18]. It has a total of 29 items on a 4-point Likert scale. Each statement contains alternatives about a potential stigma issue. Participants reply to each statement by indicating their level of agreement: (1) for strongly disagree; (2) for disagree; (3) for agree; and (4) for strongly agree. It contains five subscales; Alienation (6 items), Stereotype Endorsement (7 items), Discrimination Experience (5 items), Social Withdrawal (6 items), and Stigma Resistance (5 items). Except for stigma resistance domain, a higher score of the remaining four subscales indicates higher internalized stigma. For that matter, stigma resistance items were reverse-coded. The overall score was obtained by summing all the answered scores and divided by a total number of items.

Additionally, a semistructured questionnaire was used to collect background information. Perceived social support was assessed by using semistructured and open-ended questions. Participants' clinical characteristics such as diagnosis, duration of illness, treatment, medication side effects, and other clinical factors were collected from patients' charts and from their self-report.

High internalized stigma is defined as a score of 2.5 and above among the four Likert scale questions of ISMI. Low internalized stigma refers to participants who scored below 2.5 points of ISMI. Comorbidity defined as a situation in which physical illness occurs simultaneously with mental illness.

### 2.4. Instrument Adaptation Procedure

The questionnaire was translated to Amharic and then back-translated to English. After we checked its consistency with its original English version, the tool was back-translated into Amharic. Furthermore, the questionnaire was pretested before its duplication preliminary. Data collectors were trained for two consecutive days on interview technique and ethical issues. The training aimed to address how to maintain confidentiality, how to keep the quality of data, and how to minimize underreporting by study participants. Close supervision was made during the data collection. The collected data were cross-checked for consistency and completeness each day.

## 3. Statistical Analysis

The collected data were double entered using EPI data, software version 3.1. Then it was exported to SPSS version 20.0 for data processing and analysis. Descriptive tests like proportion, mean, and standard deviations were calculated. Taking the first categorical response option as a reference, binary logistic regression analysis was done. Hosmer-Lemeshow goodness of fit was used to test model fitness. Odds ratio along with the 95% CI was estimated to ascertain the association between covariates and internalized stigma. Covariates that have *P* value of <0.25 at the bivariate logistic regression were selected. Then the selected variables were analyzed once with the outcome variable to control all possible confounding factors. Those factors which showed a *P* value ≤ 0.05 at multivariate logistic regression were assumed as statistical significance.

### 3.1. Ethical Approval

An officially written approval letter was obtained from Research and Dissemination Office of Dilla University prior to the study. Written permission letter was obtained from the DURH clinical director and the psychiatry clinic. Oral informed consent was also obtained from each study participant. The interviewers were told to inform each respondent about all details of the research, what is expected of them, and the way information will be handled. Confidentiality of their responses was assured by interviewing them privately and by coding their name.

## 4. Results

### 4.1. Sociodemographic and Clinical Characteristics

Of the total 317 respondents, 56.5% (*n* = 119) were urban residents with a mean age of 33.6 years (standard deviation of 9.59 years). The majority of responders (69.4%, (*n* = 220)) were male. The data also revealed that 23.6% (*n* = 75) of the participants were illiterate. More than one-third, 38.1% (*n* = 121) of the study participants were farmers. Around one-fifth, 18.4% (*n* = 58) of the study participants reported unemployment. Half of the study participants, 51.3% (*n* = 163), were married. Sociodemographic characteristics are illustrated in (Table 1). Forty percent (*n* = 129) of the participants experienced different forms of medication side effects at some time. The result is well described in Tables 2 and 3.

From the study participants, 32.1% had a high level of self-stigma based on total stigma score. The mean for the overall self-stigma score was 2.36. High self-stigma with respect to alienation (36.4%), stigma endorsement (29.9%), and societal discrimination (35.2%) was found.

The majority of patients were diagnosed with psychosis (52.05%). The mean duration of the patient's mental illness was 4.37 years (SD = 2.83) while the mean of time since the initiation of psychiatric follow-up was 3.43 years (SD = 2.26) (Table 3).

## 5. ISMI Factor Domains

### 5.1. Internalized Stigma with respect to Alienation

Out of a four-point scale, the mean alienation (feeling of being inferior) score was 2.32 (SD = 0.40). Around 36% of the study participants have a high score of alienation. Females had significantly higher alienation (AOR = 0.11, CI (0.09, 0.65), *P* < 0.05) than males. Education level was positively correlated with alienation (AOR = 0.45, CI (0.11, 0.82), *P* < 0.01). As alienation score raised, treatment adherence decreases significantly (AOR = 0.45, CI (0.67, 0.95), *P* < 0.05).

### 5.2. Internalized Stigma with respect to Stereotype Endorsement

The mean score for agreeing on the common stereotypes about people with a mental illness was 2.20 (SD = 0.24). Around 30% of the study participants had the highest score of stigma endorsement. Those study participants with no family support were experiencing higher stereotype than those with good family support (AOR = 3.12, CI (2.21, 4.53), *P* < 0.005).

### 5.3. Internalized Stigma with respect to Discrimination Experience

The mean perceived discrimination score was 2.27 (SD = 0.34). Significant association was identified between educational status and a high score of discrimination experience (AOR: 0.92, CI (0.22, 0.99), *P* < 0.05).

### 5.4. Internalized Stigma with respect to Social Withdrawal

Almost 37% of the study participants showed high scores of societal withdrawal. However significant association was not found between independent variables and self-stigma with respect to social withdrawal.

### 5.5. Internalized Stigma with respect to Stigma Resistance

The mean score for stigma resistance subscale was 2.20 (SD = 0.34). Forty percent of study participants' experienced the lowest score of stigma resistance.

### 5.6. Overall Internalized Stigma

The overall prevalence of internalized stigma among people with mental illness (PWMI) was 32.1%. Binary and multiple logistic regressions were done to identify factors affecting internalized stigma among the study population. Females were experiencing higher internalized stigma than their counterparts.  Table 4 illustrates the factors independently associated with internalized stigma.

## 6. Discussion

The overall prevalence of internalized stigma among psychiatric follow-up patients in DURH is supported by other findings in Europe, which indicated that there are rampant self-stigmatization and discrimination among people with mental illness [23, 24].

Our finding is higher compared to other study findings, 25.1% in southwest Ethiopia and 22.5% in Nigeria [8, 18]. The higher prevalence of internalized stigma identified in this study might be explained by a low level of awareness about the nature of mental illness among community dwellers in the current study area. Other reason could be that mental health professionals working in the current study are probably more focused on prescriptions, rather than providing strong psychoeducation, counseling, and psychotherapy sessions to combat internalized stigma among follow-up patients.

In our study, the prevalence of internalized stigma was lower compared to other studies' findings out of Ethiopia, 40% in Iran, 36% in California, and 35% in Maryland [5–7]. Another study finding from Addis Ababa, Ethiopia, reported that 97.4% of schizophrenia patients experienced at least one internalized stigma item [9]. Internalized stigma seems high in specific severe mental disorders. The perceived effect of stigma is greater if the patient had more prominent positive symptoms. The great subjective feeling that they are not full members of society leads to objective discrimination [24]. The self-concept about internalized stigma may differ by culture and knowledge of a specific community. Minimal and reasonable amounts of rejection may lead to internalized stigma in the western community. So that other large-scale studies are required to explain the difference in detail.

In this study, there was a positive significant correlation between internalized stigma and gender. There was evidence that suggests being female is a risk factor for the development or exacerbation of mental health conditions [25]. It is believed that females are more stigmatized than males for the same behavior. They may have limited marriage invitation by males because of their illness. Therefore, such kinds of rejection and public stigma may negatively influence internalized stigma. However several kinds of literature claimed that there is no significant association between sociodemographic variables including gender and internalized stigma [6, 15]. It is complex to set a conclusion as such and further studies must be carried out to ascertain the impact of sociodemography on internalized stigma.

According to our study, patients getting good family support had less risk for internalized stigma than their counterparts. Other previous study findings showed similar results [3, 19, 23]. These studies strongly stressed that individuals getting better social and family support had a better prognosis and good outcome against internalized stigma. Furthermore, they are actively involved in antistigma campaigns.

Study participants who adhere to their medication as ordered by clinicians were experiencing less internalized stigma than those study participants who do not take their medication properly. It is in agreement with other research outcomes [16, 26, 27]. Visiting psychiatric clinics and taking medications are directly associated with disclosure of their health status. And study participants claimed that they have poor help-seeking behavior. They also prefer stopping follow-up to escape from experiencing internalized stigma associated with their psychiatric condition.

## 7. Conclusion

This study identified an intermediate prevalence of internalized stigma among psychiatric follow-up patients in southern Ethiopia. Female gender, medication nonadherence, and inadequate family support predict internalized stigma. Strategies focusing on early detection and treatment of mental illness played important role in stigma prevention. Awareness creation, community mobilization, and service expansion strategies are cost-effective and efficient ways to act against stigma. Further, strengthening social network (support) of mentally ill people with themselves and with the community is found to be necessary. Expand training program and empower people with mental illness are expected to bring positive change. These programs should reach all social classes and cultures in schools, universities, social clubs, religious institutions, and mass media through the outreach health education program. Psychiatric institutions should do more roles relying not only on medications, but also on promotion of patients' social life. Increase patient's awareness of certain issues could promote relapse prevention, adequate social skills, and assumption of responsibility in life. Mass media should play a role in the destigmatization of psychiatric patients and psychiatric illness as well.

## Figures and Tables

**Table 1:** Sociodemographic characteristics of study participants.

Variable	Number	%
Sex		
Male	220	69.4
Female	97	39.7
Age (in years)		
18–24	67	21.0
25–34	110	34.8
35–49	140	44.3
Educational status		
Illiterate	75	23.6
Elementary	116	36.3
High school	44	14.0
College and above	82	25.9
Marital status		
Single	132	41.6
Married	151	47.5
Others	34	10.9
Religion		
Protestant	145	45.6
Orthodox	86	27.1
Others	86	27.1
Ethnicity		
Gedio	141	44.6
Sidamo	76	24
Guragie	47	14.8
Oromo	36	11.8
Income		
<500 BIRR	116	36.4
>500 BIRR	202	63.6
Occupation		
Unemployed	58	18.4
Student	27	8.4
Farmer	121	38.1
Governmental worker	111	35.1

**Table 2:** Social support characteristics of participants.

Variable	Number	%
Living status		
Alone	68	21.4
With family	221	69.6
With relatives	23	7.2
With friend	5	1.8
Family support		
Yes	257	81.1
No	60	18.9
Type of support from family		
Moral support	104	32.8
Physical support	81	25.4
Financial support	61	19.4
Food support	71	22.4
Support from society		
Yes	156	48.9
No	162	51.1
Source of societal support		
From friends	64	20.8
From relatives	42	13.3
From religion	15	4.6
From government	23	7.4
From neighbors	9	2.8

**Table 3:** Clinical characteristics of participants.

Variable	Number	%
Duration of treatment		
Less than 2 years	76	24.0
2–5 years	77	24.3
6–10 years	110	34.7
>10 years	54	17.6
Adherence to medications		
Yes	122	38.5
No	195	61.5
If not how often you miss it?		
Every 3 days	16	5.2
Every week	142	44.9
Every months	28	8.8
Others	9	2.1
Any side effect associated with medication		
Yes	205	64.6
No	112	35.4
Types of side effects		
Weight gain	88	27.9
Tremor	43	13.7
Sexual problem	59	18.5
Sedation	6	1.8
Gait abnormality	4	1.2
Others	3	0.9

**Table 4:** Multivariate logistic regression; factors associated with high stigma scores.

Variable	AOR	95% C.I	*P* value
Sex			
Male		1	
Female	0.11	0.09, 0.65	0.02
Educational status			
Illiterate		1	
Elementary	0.45	0.11, 0.82	0.001
Secondary	0.85	0.26, 0.95	0.002
College	0.92	0.22, 0.99	0.0001
Family support status			
Yes		1	
No	3.12	2.21, 4.53	0.002
Community social support status			
Yes		1	
No	2.63	1.65, 3.43	0.0001
Medication adherence status			
Yes		1	
No	0.45	0.67, 0.95	0.03
Experiencing medication side effect			
Yes		1	
No	0.32	0.2, 0.5	0.006

*Key*. AOR = adjusted odds ratio, CI = confidence interval.

## Abbreviations

QoL: Quality of life
PWMI: People with mental illness
WHO: World Health Organization
DALY: Disability adjusted life years
DURH: Dilla University Referral Hospital
ISMH: Internalized stigma measuring tool.

## Additional Points

*Availability of Data and Materials*. The data that support the findings of this study have a sort of identifier of individual participants and researcher reserved to it.

## References

[1] Daneil E., Marilyn F., Efra G. (2009). Stigma and Help seeking for mental health among college students. *Medical Care Research and Review*.

[2] Patrick J. M., Marcelino L., Nicolas R. (2012). Constructs and concepts comprising the stigma of mental illness. *Psychology, Sociology and Education*.

[3] World Health Organization (2002). *Reducing Stigma and Discrimination against Older People with Mental Disorders*.

[4] World Health Organization Depression as a global crisis, World mental health day.

[5] Ghanean H., Nojomi M., Jacobsson L. (2011). Internalized stigma of mental illness in Tehran, Iran. *Stigma Research and Action*.

[6] West M. L., Yanos P. T., Smith S. M., Roe D., Lysaker P. H. (2011). Prevalence of Internalized Stigma among Persons with Severe Mental Illness. *Stigma Research and Action*.

[7] Amy L., Alicia L., Paul B. (2013). A model of internalized stigma and its effects on people with mental illness. *Psychiatric Services*.

[8] Ibrahim A. W., Mukhtar Y., Sadique P. (2016). A facility-based assessment of internalized stigma among patients with severe mental illnesses in Maiduguri, North-Eastern Nigeria. *International Neuropsychiatric Disease Journal*.

[9] Assefa D., Shibre T., Asher L., Fekadu A. (2012). Internalized stigma among patients with schizophrenia in Ethiopia: a cross-sectional facility-based study. *BMC Psychiatry*.

[10] Anneli P. (2010). *Improving Quality of Life of Patients with Schizophrenia*.

[11] World Health Organization (2001). *Mental Health and Stigma, A Call for Action by World Health Ministries*.

[12] Batinic B., Lemonis E., Opacic G. (2014). Effects of internalized stigma of mental disorder on quality of life and selfesteem in panic disorder patients. *Journal of Clinical Research and Bioethics*.

[13] Karakas S. A., Okanlı A., Yılmaz E. (2016). The effect of internalized stigma on the self esteem in patients with schizophrenia. *Archives of Psychiatric Nursing*.

[14] Girma E., Möller-Leimkühler A. M., Dehning S., Mueller N., Tesfaye M., Froeschl G. (2014). self-stigma among caregivers of people with mental illness: toward caregivers' empowerment. *Journal of Multidisciplinary Healthcare*.

[15] Livingston J. D., Boyd J. E. (2010). Correlates and consequences of internalized stigma for people living with mental illness: a systematic review and meta-analysis. *Social Science & Medicine*.

[16] Aphroditiz S., Michael M. (1998). *Stigma Related to Help Seeking for Mental Health Professionals*.

[17] Reta Y., Tesfaye M., Girma E., Dehning S., Adorjan K. (2016). Public stigma against people with mental illness in Jimma Town, Southwest Ethiopia. *PLoS ONE*.

[18] Girma E., Tesfaye M., Froeschl G., Möller-Leimkühler A. M., Dehning S., Müller N. (2013). Facility based cross-sectional study of self stigma among people with mental illness: Towards patient empowerment approach. *International Journal of Mental Health Systems*.

[19] Faith B. D., Jawel S., Andrea E. (2002). Experiences of stigma among outpatients with schizophrenia. *Schizophrenia Bulletin*.

[20] Girma E., Tesfaye M., Froeschl G., Möller-Leimkühler A. M., Müller N., Dehning S. (2012). Public stigma against people with mental illness in the Gilgel Gibe Field Research Center (GGFRC) in Southwest Ethiopia. *PLoS ONE*.

[21] Wodegiworgise T., Simme K. (2012). *National Mental Health Strategy*.

[22] Ritsher J. B., Otilingam P. G., Grajales M. (2003). Internalized stigma of mental illness: Psychometric properties of a new measure. *Psychiatry Research*.

[23] David M. D. Countering the stigmatization and discrimination of people with mental health problems in Europe. http://ec.europa.eu/health/archive/ph_determinants/life_style/mental/docs/stigma_paper_en.pdf.

[24] Siobhan P. (2012). How does stigma affect people with mental illness?. *Nursing Times*.

[25] Al-Krenawi A., Graham J. R., Kandah J. (2000). Gendered utilization differences of mental health services in Jordan. *Community Mental Health Journal*.

[26] Ehab S. R., Wessam A. E. (2010). Relation between insight and quality of life in patients with schizophrenia. *Current Psychiatry*.

[27] Ayenalem E. A., Tiruye T. Y., Muhammed M. S. (2017). Impact of self stigma on quality of life of people with mental illness at dilla university referral hospital, South Ethiopia. *American Journal of Health Research*.

